# Decoding neuroimmune ferroptotic vulnerability in isoflurane-induced neonatal neurotoxicity via the SLC7A11/GPX4 axis

**DOI:** 10.3389/fphar.2026.1803172

**Published:** 2026-06-02

**Authors:** Pingping Huang, Chengyu Chen, Mengcong Wang, Lijun Xue

**Affiliations:** Department of Anesthesiology, The First Affiliated Hospital of Wenzhou Medical University, Wenzhou, Zhejiang, China

**Keywords:** astrocytes, ferroptosis, isoflurane, neonatal neurotoxicity, neuroimmune remodeling, SLC7A11/GPX4 axis

## Abstract

**Introduction:**

Early-life exposure to general anesthetics has been associated with increased neurodevelopmental vulnerability, but the cell type-resolved mechanisms remain incompletely understood. Here, we investigated whether isoflurane-induced neonatal neurotoxicity is associated with a glia-linked ferroptotic susceptibility state involving altered SLC7A11/GPX4 antioxidant defense signaling.

**Methods:**

By integrating single-cell and bulk transcriptomic profiling with *in vivo* behavioral, histological, and biochemical phenotyping, we identified glia-enriched transcriptional changes associated with inflammatory activation, impaired cystine-glutathione metabolism, and increased lipid peroxidation. In primary neonatal astrocytes, isoflurane induced ferroptosis-related phenotypes accompanied by axis suppression, redox imbalance, and lipid peroxidation, which were partially attenuated by pharmacological rescue. Additional inflammatory challenge and neutralization experiments further showed that immune-associated signaling functionally contributes to suppression of the astrocytic SLC7A11/GPX4 defense axis. Pathway-comparison rescue experiments further indicated that ferroptosis-directed intervention more effectively improved lipid peroxidation-associated injury readouts, whereas apoptosis-directed intervention more strongly reduced caspase-associated changes.

**Results:**

Isoflurane exposure was accompanied by neurodevelopmental and behavioral impairments, reduced expression of SLC7A11/GPX4 axis components, increased lipid oxidative damage, and enhanced neuroimmune reactivity, with convergent data suggesting that astrocytes are an important cellular contributor to this injury-associated state.

**Discussion:**

Together, these findings support a glia-associated neuroimmune ferroptotic vulnerability framework in isoflurane-induced neonatal neurotoxicity and identify the SLC7A11/GPX4 axis as a candidate mechanism warranting further causal investigation in peri-anesthetic neuroprotection for the developing brain.

## 1 Introduction

In contemporary pediatric practice, a growing number of neonates and infants undergo surgical procedures for congenital or acquired conditions. Isoflurane, a commonly used inhalational anesthetic, plays an important role in maintaining procedural safety and physiological stability during surgery (Hu et al., 2024). At the same time, accumulating clinical and experimental evidence has raised concern that prolonged or repeated anesthetic exposure during early life may increase the risk of adverse neurodevelopmental outcomes (Miao et al., 2024). These concerns are especially relevant to cognitive and behavioral domains, including learning and memory, which may be vulnerable during critical periods of brain maturation. Although regulatory agencies have issued warnings regarding the potential neurodevelopmental risks associated with repeated or prolonged general anesthesia in children younger than 3 years, the biological basis of anesthesia-related developmental neurotoxicity remains incompletely understood, and effective neuroprotective strategies remain limited (Zhang P. et al., 2024; Gu et al., 2025).

Ferroptosis is a regulated cell-death process characterized by iron-dependent lipid peroxidation and failure of cellular redox defense. Emerging evidence suggests that ferroptosis-related processes may contribute to perioperative and anesthesia-associated brain injury, including isoflurane-related neurotoxicity in the developing brain (Fan et al., 2024). Previous studies have shown that isoflurane exposure can be associated with iron accumulation, glutathione depletion, and lipid peroxide generation in immature brain tissue, particularly in regions with high developmental metabolic demand. Within this context, the SLC7A11/GPX4 axis represents an important antioxidant defense pathway. SLC7A11 supports cystine uptake for glutathione synthesis, whereas GPX4 detoxifies lipid hydroperoxides and helps limit oxidative membrane damage (Zhang C. et al., 2024). Disruption of this axis may weaken redox buffering capacity and increase vulnerability to lipid peroxidation-associated injury.

Importantly, ferroptosis-related vulnerability in the developing brain is unlikely to occur in isolation and may be shaped by the neonatal neuroimmune microenvironment (Lao et al., 2026). During early postnatal development, microglia participate in synaptic refinement and circuit maturation, and their functional state is sensitive to environmental perturbation. Isoflurane exposure has been reported to induce neuroimmune activation, including changes involving microglia and inflammatory cytokine signaling (Zuo et al., 2025). Such immune-associated responses may influence local iron handling, glutathione metabolism, and oxidative balance, thereby lowering the threshold for ferroptosis-related injury. In neonatal neural tissue, which is enriched in polyunsaturated lipids and has relatively limited antioxidant reserve, this neuroimmune-associated redox disturbance may further amplify lipid oxidative damage and interact with other injury pathways, including apoptosis-associated signaling (You et al., 2024).

Although previous studies have described neuroinflammatory responses and ferroptosis-related alterations in anesthesia-associated brain injury, the relationship between these processes and the involvement of the SLC7A11/GPX4 axis remain insufficiently defined (Tang et al., 2025). In particular, it remains unclear how glial and neuroimmune responses reshape the neonatal brain microenvironment in ways that may influence ferroptosis susceptibility after isoflurane exposure, and whether immune-associated signaling functionally contributes to suppression of astrocytic antioxidant defense. To address this gap, we established a neonatal mouse model of isoflurane exposure and integrated single-cell and bulk transcriptomic analyses with *in vivo* and *in vitro* validation to examine neuroimmune-associated ferroptotic susceptibility. We focused on glia-enriched transcriptional changes, with particular attention to astrocyte-associated alterations involving the SLC7A11/GPX4 axis, and evaluated whether inflammatory challenge and neutralization, together with pathway-comparison rescue experiments, could clarify the contribution of immune-associated signaling and distinguish ferroptosis-related injury from coexisting apoptosis-associated changes (Huang et al., 2025).

## 2 Materials and methods

### 2.1 Study design and animals

A randomized, controlled, parallel intervention-and-rescue design was used to examine isoflurane-induced neonatal neurotoxicity, ferroptosis-associated vulnerability, and the partial reversibility of injury-related molecular and phenotypic changes in the developing brain. The study was designed to evaluate the involvement of the SLC7A11/GPX4 antioxidant defense axis using integrated *in vivo*, transcriptomic, and *in vitro* validation modules. C57BL/6J mice were used for all experiments, and postnatal day 7 (P7) was selected as the primary exposure time point because it represents a developmentally vulnerable stage of rapid brain maturation.

To minimize intra-litter variability, stratified randomization was performed within each litter, and pups were distributed across groups according to body weight and sex, with animals from the same litter represented in each experimental group (Li ChangMD. et al., 2025). Blinding was implemented for histological quantification, image analysis, and molecular readout processing. Sample identifiers were coded by personnel not involved in downstream analyses and were unblinded only after completion of primary statistical testing.

All animal procedures complied with institutional animal care and use regulations and were approved by the relevant ethics committee. Maternal separation time was minimized, and all exposure and tissue collection procedures were conducted under controlled temperature conditions to reduce nonspecific stress-related confounding.

### 2.2 Isoflurane exposure and monitoring

Isoflurane exposure was performed in a sealed anesthesia chamber. P7 pups were placed in a preheated chamber immediately after separation from the dam, and chamber temperature was maintained at 37 °C ± 0.5 °C throughout the exposure period. Isoflurane was delivered at 1.5% for 120 min using a carrier gas mixture of oxygen and nitrogen, with the oxygen concentration maintained at 30%–50%. Control animals were placed in the same chamber under identical temperature and carrier gas conditions without isoflurane exposure.

During exposure, respiratory rate, skin color, and spontaneous activity were monitored at 15–20 min intervals to assess physiological stability. Exposure was terminated immediately if persistent respiratory depression, cyanosis, or signs of acute distress were observed, and such animals were excluded according to predefined criteria. After exposure, pups were rewarmed on a temperature-controlled mat and returned to the dam after recovery of spontaneous activity, with maternal separation kept to the minimum necessary duration.

### 2.3 Interventions and experimental groups

Four *in vivo* experimental groups were established to assess baseline phenotypes, ferroptosis-associated rescue effects, and axis-supportive intervention responses: (1) control (carrier gas only), (2) isoflurane (1.5% for 2 h), (3) isoflurane plus Ferrostatin-1 (Fer-1), and (4) isoflurane plus axis-supportive intervention (N-acetylcysteine plus selenium).

Ferrostatin-1 was administered intraperitoneally at 5 mg/kg. The first dose was given 30 min before isoflurane exposure to cover the early phase of lipid peroxidation initiation, and a second dose was given 24 h after exposure to target ongoing lipid radical propagation during injury progression. This regimen was used as a pharmacological rescue strategy to evaluate whether attenuation of ferroptosis-associated lipid peroxidation was accompanied by improvement in downstream injury phenotypes.

The axis-supportive intervention consisted of combined N-acetylcysteine (NAC) and selenium supplementation, selected to support cystine/glutathione availability and GPX4-related antioxidant function. NAC was administered intraperitoneally at 150 mg/kg using the same dosing schedule as Fer-1. Selenium was administered intraperitoneally at 0.2 mg/kg 30 min before exposure and then daily for 2 days after exposure (0.2 mg/kg per dose) to support selenoprotein-dependent antioxidant activity, including GPX4. Isovolumetric solvent controls were used to standardize injection volume and handling stress across groups.

Because NAC and selenium have biological effects beyond the SLC7A11/GPX4 axis, this intervention was interpreted as an axis-supportive rather than axis-specific rescue strategy. Therefore, findings from this group were used to evaluate whether enhancement of antioxidant capacity aligned with recovery of axis-related markers and ferroptosis-associated phenotypes, rather than as definitive proof of axis-specific causality.

To reduce bias related to litter effects, pups were drawn from multiple litters, and litter was considered a potential clustering factor in statistical models when appropriate. Primary intervention outcomes included axis-related molecular expression, lipid peroxidation, iron homeostasis, and glia-associated reactivity readouts. Additional pathway-discriminating and inflammatory challenge experiments were performed in the *in vitro* astrocyte module as described in Section 2.8.

### 2.4 Tissue collection and histology

Tissue collection was performed primarily at 24 h after exposure. Neonatal mice were deeply anesthetized with isoflurane (3%–4% in oxygen) until complete loss of pedal withdrawal and corneal reflexes. For molecular and biochemical assays, animals were euthanized by rapid decapitation under deep isoflurane anesthesia, and brain tissues were harvested immediately to minimize ischemia-related artifacts. For histological and immunofluorescence analyses, animals remained under deep isoflurane anesthesia and were transcardially perfused with cold phosphate-buffered saline (PBS), followed by 4% paraformaldehyde (PFA) for fixation. All procedures were conducted in accordance with approved institutional animal care protocols.

Brain tissue was processed using two parallel workflows. For molecular and biochemical assays, hippocampi and cortices were rapidly dissected on ice, snap-frozen in liquid nitrogen, and stored at −80 °C for subsequent immunoblotting, qPCR, and measurements of glutathione, malondialdehyde (MDA), and ferrous iron. For histology and immunofluorescence, brains collected after perfusion were removed carefully and post-fixed in 4% PFA at 4 °C, followed by standardized dehydration and embedding procedures. In animals not subjected to perfusion-based fixation, whole brains were rapidly removed and immersion-fixed under matched fixation conditions. Fixation conditions were kept consistent across groups to preserve tissue morphology and antigenicity.

Histological analyses focused on neurodegeneration-associated changes, glia-associated reactivity, and ferroptosis-related lipid peroxidation. Neurons, astrocytes, and microglia were identified using NeuN, GFAP, and Iba1 staining, respectively. Iba1 immunostaining was used to assess overall microglia/macrophage-associated reactivity and morphology and was not intended to provide definitive resolution of fine-grained microglial activation states. SLC7A11 and GPX4 expression was assessed by immunostaining and/or tissue lysate analyses, and lipid peroxidation was evaluated using 4-hydroxynonenal (4-HNE) as a core marker. Immunofluorescence quantification followed a standardized sampling workflow, with sections selected from comparable anatomical levels and multiple fields analyzed per section. Image acquisition parameters were held constant across groups, and image analysis was performed in a blinded manner.

To support neuroimmune-associated interpretation, histological analysis also examined the spatial distribution of 4-HNE and GPX4 immunoreactivity relative to glial marker-positive regions. These observations were interpreted cautiously as descriptive histological correlates rather than definitive spatial-causal evidence and were integrated with molecular and biochemical readouts to characterize injury burden and redox disruption after isoflurane exposure.

### 2.5 Molecular and biochemical assays

A combined molecular and biochemical workflow was used to evaluate antioxidant defense, iron homeostasis, and lipid peroxidation after isoflurane exposure. Frozen hippocampal and cortical tissues were processed in parallel batches to reduce inter-assay variability. Protein extraction was performed using RIPA buffer supplemented with protease and phosphatase inhibitors, followed by homogenization and protein quantification. Equal amounts of protein were loaded for immunoblotting of SLC7A11, GPX4, ACSL4, TFRC, FTH1, and PTGS2. HO-1 and 4-HNE adducts were measured as supplementary oxidative stress and lipid peroxidation indicators when applicable. GAPDH or β-actin was used as an internal loading control. Technical repeat runs were performed when necessary to verify signal stability.

For mRNA analysis, total RNA was extracted and reverse-transcribed, and gene expression was quantified by real-time qPCR using consistent primer sets and reaction conditions across samples. Relative expression was calculated using the 2^−ΔΔCt^ method and compared with corresponding protein-level trends for internal consistency.

Biochemical assays focused on ferroptosis-relevant parameters. Total glutathione and GSH/GSSG ratios were measured using commercial kits to assess redox reserve. Lipid peroxidation was quantified by MDA and interpreted together with 4-HNE measurements to provide complementary biochemical and molecular evidence. Ferrous iron content was measured as an indicator of iron load and integrated with TFRC and FTH1 expression data. All biochemical readouts were normalized to protein concentration or tissue weight, and matched tissue blocks were used whenever possible to facilitate cross-modality comparisons.

### 2.6 scRNA-seq and bulk RNA-seq

Single-cell transcriptomic profiling was performed to characterize cell type-resolved neuroimmune remodeling and ferroptosis-associated vulnerability after isoflurane exposure. Samples were collected primarily at 24 h post-exposure. For the single-cell workflow, hippocampal and cortical tissues were pooled within each biological replicate to improve cell yield and preserve broad representation of developmentally vulnerable brain cell populations. Because this approach prioritizes cell recovery and atlas stability over regional resolution, downstream single-cell findings were interpreted as cross-region trends rather than region-specific responses.

Tissues were dissociated using gentle enzymatic digestion with DNase to reduce aggregation and free-DNA contamination, followed by filtration through a 40 μm mesh. Debris-removal procedures were applied to reduce myelin and necrotic material, and only viable cells passing predefined quality thresholds were used for library construction.

Single-cell libraries were generated using a droplet-based platform, targeting approximately 8,000–12,000 cells per sample. Sequencing depth was adjusted according to cell number and transcriptomic complexity to ensure adequate coverage of both glial and neuronal populations. Data processing included quality-control filtering, normalization, batch correction, dimensionality reduction, clustering, and marker-based annotation. Cell types and broad glia-associated transcriptional states were annotated using established marker genes and reference-based cross-checking, with particular attention to astrocyte and microglia-enriched populations.

A ferroptosis-related susceptibility score was calculated at the cell-population level using a curated gene set including SLC7A11, GPX4, ACSL4, ALOX family members, FTH1, and TFRC. This score was used as a comparative transcriptomic indicator of ferroptosis-associated vulnerability rather than as a standalone determinant of cell-death mechanism. Differential expression and pathway enrichment analyses were then performed between control and isoflurane-exposed groups within major cell populations.

Bulk RNA-seq was performed on matched time points and corresponding brain tissue samples to validate and extend single-cell observations at the tissue level. Differential expression analyses focused on antioxidant metabolism, iron homeostasis, lipid metabolism, and inflammatory signaling pathways. Bulk transcriptomic data were used to complement single-cell findings and to support distinction between cell composition shifts and intracellular transcriptional reprogramming.

### 2.7 Multi-omics integration

Multi-omics integration was conducted to align cell type–resolved transcriptional features with tissue-level transcriptomic modules and phenotypic readouts. Cell-type signatures derived from scRNA-seq were projected onto bulk RNA-seq patterns to assess the relative contribution of cell-state shifts and population-level changes. Pathway enrichment and module-based analyses were used to identify transcriptomic modules associated with ferroptosis, glutathione metabolism, and inflammatory stress.

Correlation analyses were performed between transcriptomic modules and experimental phenotypes, including lipid peroxidation burden, iron load, and axis-related molecular expression. To prioritize candidate regulatory nodes, network-level analyses considered differential expression strength, connectivity, and contribution to ferroptosis-related susceptibility scores across major glial populations. This integrative approach was used to identify SLC7A11/GPX4-associated modules and to guide downstream validation and intervention analyses.

### 2.8 *In vitro* astrocyte model and rescue tests

*In vitro* experiments using primary neonatal astrocytes were performed to validate astrocyte-associated ferroptosis-related responses under controlled conditions and to test pharmacological rescue effects. Primary astrocytes were isolated from the cortex and/or hippocampus of P1–P3 neonatal mice by mechanical and enzymatic dissociation and cultured in coated flasks in serum-containing medium. Non-astrocytic cells were reduced by shaking enrichment to improve astrocyte purity. After stabilization in culture, cells were subjected to experimental treatments.

Where applicable, isoflurane exposure was delivered using a sealed volatile anesthetic exposure system under temperature-controlled conditions aligned as closely as possible with the *in vivo* paradigm. Cells were then returned to standard culture conditions, and samples were collected at predefined time points for molecular and functional analyses.

Ferroptosis-related phenotypes were evaluated using multiple readouts, including lipid ROS (BODIPY-C11), ferrous iron assays, glutathione and GSH/GSSG measurements, and cell viability assays (e.g., CCK-8). Expression of SLC7A11, GPX4, ACSL4, and PTGS2 was assessed by immunoblotting and/or immunofluorescence.

For baseline rescue experiments, Ferrostatin-1 was used as a pharmacological rescue control for ferroptosis-associated lipid peroxidation and was administered before exposure and maintained during the relevant post-exposure window. Axis-supportive intervention included NAC and selenium supplementation to evaluate whether strengthening antioxidant capacity was associated with recovery of SLC7A11/GPX4-related readouts and attenuation of ferroptosis-like phenotypes.

To further examine whether immune-associated signaling functionally contributes to suppression of the astrocytic SLC7A11/GPX4 defense axis and to compare ferroptosis-related and apoptosis-related injury components, an additional inflammatory challenge and pathway-comparison rescue module was performed. In this module, primary astrocytes were assigned to the following groups: (1) control, (2) isoflurane, (3) isoflurane plus TNF-α/IFN-γ, (4) isoflurane plus Ferrostatin-1, (5) isoflurane plus zVAD-fmk, (6) isoflurane plus Ferrostatin-1 plus zVAD-fmk, and (7) isoflurane plus TNF-α/IFN-γ plus inflammatory neutralization treatment.

Recombinant TNF-α and IFN-γ were used to model inflammatory cues suggested by the transcriptomic and tissue-level neuroimmune analyses. Cytokines were added during the early post-exposure window to test whether inflammatory stimulation would further aggravate axis suppression, redox imbalance, and lipid peroxidation-associated injury in astrocytes. For inflammatory neutralization, cells were treated with cytokine-neutralizing antibodies and/or matched inhibitory reagents directed against TNF-α and IFN-γ signaling, using parallel vehicle controls. These conditions were used to evaluate whether blockade of immune-associated signaling could partially restore SLC7A11/GPX4-related antioxidant defense readouts.

To distinguish ferroptosis-related from apoptosis-related injury contributions, zVAD-fmk was used as a caspase-directed apoptosis inhibitor and was applied in parallel with Ferrostatin-1. Ferrostatin-1 was interpreted primarily as an inhibitor of lipid peroxidation-associated ferroptotic injury, whereas zVAD-fmk was interpreted as an inhibitor of caspase-dependent apoptotic signaling. The combined treatment group was included to test whether simultaneous suppression of both pathways would provide greater overall rescue than either intervention alone.

In this expanded *in vitro* module, outcome measures included SLC7A11, GPX4, ACSL4, and PTGS2 expression; lipid ROS; 4-hydroxynonenal (4-HNE); glutathione-related readouts; ferrous iron; cleaved caspase-3; cleaved PARP1; and cell viability. These assays were selected to determine whether inflammatory signaling preferentially intensified ferroptosis-associated redox failure, whether apoptosis-associated changes co-occurred in parallel, and whether rescue patterns differed between ferroptosis-directed and apoptosis-directed interventions.

No genetic gain- or loss-of-function experiments were performed in the present study. Therefore, causal inference regarding the SLC7A11/GPX4 axis was interpreted cautiously and based primarily on convergent transcriptomic, molecular, biochemical, and pharmacological evidence, with the additional *in vitro* inflammatory challenge and pathway-comparison experiments providing functional support rather than definitive genetic proof of causality.

### 2.9 Statistics and omics quality control

Primary endpoints were predefined as SLC7A11/GPX4 axis-related molecular indicators and ferroptosis-associated injury readouts, whereas secondary endpoints included glia-associated reactivity markers and omics-derived transcriptomic module signals. Statistical analyses were prespecified according to data type and experimental design.

For continuous variables, normality was assessed using the Shapiro–Wilk test, and homogeneity of variance was evaluated using Levene’s test. Normally distributed data are presented as mean ± SD and were analyzed using one-way or two-way analysis of variance (ANOVA), as appropriate. When omnibus ANOVA results were significant, *post hoc* multiple-comparison testing was performed using Tukey’s test (for equal variances) or Games–Howell testing (for unequal variances), as applicable. Non-normally distributed data are presented as median (interquartile range) and were analyzed using Kruskal–Wallis tests followed by Dunn’s multiple-comparison correction.

For time-dependent outcomes, two-way models were used to evaluate the main effects of group and time and their interaction. When repeated measurements were collected from the same animal, repeated-measures ANOVA or mixed-effects models were applied depending on data structure and model fit. Effect sizes and 95% confidence intervals were reported for principal comparisons where appropriate.

To account for potential litter-related clustering, pups were sampled from multiple litters and litter was incorporated as a random effect (or nested clustering factor) in mixed-effects analyses when applicable. Sex was included as a covariate in prespecified models to reduce sex-related confounding. Unless otherwise stated, all tests were two-tailed, and P < 0.05 was considered statistically significant after correction for multiple comparisons where applicable.

For the expanded *in vitro* astrocyte experiments, including inflammatory challenge, inflammatory neutralization, and pathway-comparison rescue conditions, groupwise comparisons were performed using one-way ANOVA followed by Tukey’s *post hoc* test when a single endpoint was analyzed at one time point. When treatment structure included both isoflurane exposure and rescue/intervention factors, two-way ANOVA was used where appropriate to test main effects and interactions. Exact biological replicate numbers for each assay are reported in the corresponding figure legends. *In vitro* rescue patterns were interpreted comparatively across endpoint classes, with lipid peroxidation-, redox-, and iron-related readouts considered ferroptosis-associated indicators, and cleaved caspase-3 and cleaved PARP1 considered apoptosis-associated indicators.

For transcriptomic analyses, differential expression and pathway enrichment were performed using standard pipelines with false discovery rate (FDR) correction (Benjamini–Hochberg method) for multiple testing. Adjusted P values (FDR q values) were used for significance interpretation in omics analyses unless otherwise specified.

Omics quality control (QC) was conducted before downstream integrative analyses to ensure data reliability and comparability across groups. For scRNA-seq, low-quality cells were excluded using predefined thresholds for detected genes, UMI counts, and mitochondrial transcript proportion, and putative doublets were identified and removed prior to normalization, batch correction, clustering, and annotation. For bulk RNA-seq, QC metrics included RNA integrity (RIN), read quality (Q30), alignment rate, rRNA contamination rate, duplication rate, and detected gene counts to confirm sequencing quality and suitability for differential expression and module-based analyses.

To provide transparent documentation of dataset quality, key QC metrics for the single-cell and bulk transcriptomic datasets are summarized in Tables 1A, B, respectively. Table 1A reports post-filtering scRNA-seq quality indicators, including cells retained, gene/UMI complexity, mitochondrial reads, doublet rate, and sequencing saturation. Table 1B reports bulk RNA-seq library-level QC indicators, including RIN, raw reads, Q30, mapping rate, rRNA rate, duplication rate, and detected genes. Sample identifiers were verified and standardized across groups before analysis. The pooled “Cortex + Hippocampus” label in Table 1 indicates combined tissue processing within each biological replicate for transcriptomic profiling and should be interpreted as a cross-region sampling strategy rather than region-specific sequencing.

**TABLE 1:** Quality control metrics for scRNA-seq and bulk RNA-seq datasets.

A. Quality control metrics for scRNA-seq libraries									
Sample ID	Group	Brain region	Cells retained (n)	Median genes per cell	Median UMIs per cell	Mito reads (%)	Doublet rate (%)	Mean reads per cell	Sequencing saturation (%)
scCTRL_1	Control	Cortex + hippocampus	9,842	1,740	5,980	6.1	3.8	48,600	73
scCTRL_2	Control	Cortex + hippocampus	10,126	1,812	6,210	5.7	4.1	50,200	74
scCTRL_3	Control	Cortex + hippocampus	9,515	1,695	5,720	6.4	3.6	47,900	72
scISO_1	Isoflurane	Cortex + hippocampus	9,106	1,632	5,340	7.8	4.2	49,100	72
scISO_2	Isoflurane	Cortex + hippocampus	9,488	1,668	5,510	7.2	4.0	50,400	73
scISO_3	Isoflurane	Cortex + hippocampus	8,934	1,601	5,180	8.0	4.4	48,700	71
scISO_Fer1_1	ISO + Fer-1	Cortex + hippocampus	9,372	1,701	5,760	6.6	3.9	49,800	73
scISO_Fer1_2	ISO + Fer-1	Cortex + hippocampus	9,640	1,745	5,920	6.3	4.0	50,900	74
scISO_Fer1_3	ISO + Fer-1	Cortex + hippocampus	9,214	1,688	5,650	6.7	4.1	49,300	73
scISO_Axis_1	ISO + axis-supportive	Cortex + hippocampus	9,558	1,776	6,030	6.2	3.7	50,600	74
scISO_Axis_2	ISO + axis-supportive	Cortex + hippocampus	9,901	1,823	6,240	5.9	3.8	51,100	75
scISO_Axis_3	ISO + axis-supportive	Cortex + hippocampus	9,307	1,751	5,980	6.1	3.9	49,700	74

B. Quality control metrics for bulk RNA-seq libraries									
Sample ID	Group	Brain region	RIN	Raw reads (M, PE)	Q30 (%)	Mapping rate (%)	rRNA rate (%)	Duplication (%)	Detected genes (n)
bulkCTRL_1	Control	Cortex + hippocampus	8.6	48.2	92.4	95.1	1.3	18.9	16,820
bulkCTRL_2	Control	Cortex + hippocampus	8.4	50.6	92.1	94.6	1.5	19.6	16,940
bulkCTRL_3	Control	Cortex + hippocampus	8.7	46.9	92.8	95.4	1.2	18.1	16,770
bulkISO_1	Isoflurane	Cortex + hippocampus	8.3	49.7	92.0	94.2	1.6	20.8	16,540
bulkISO_2	Isoflurane	Cortex + hippocampus	8.2	47.8	91.7	93.9	1.8	21.4	16,460
bulkISO_3	Isoflurane	Cortex + hippocampus	8.5	51.1	92.3	94.7	1.4	20.1	16,610
bulkISO_Fer1_1	ISO + Fer-1	Cortex + hippocampus	8.6	48.9	92.6	95.0	1.3	19.2	16,720
bulkISO_Fer1_2	ISO + Fer-1	Cortex + hippocampus	8.5	46.5	92.2	94.8	1.4	19.7	16,680
bulkISO_Axis_1	ISO + axis-supportive	Cortex + hippocampus	8.7	49.4	92.9	95.3	1.2	18.6	16,850
bulkISO_Axis_2	ISO + axis-supportive	Cortex + hippocampus	8.6	47.2	92.5	95.0	1.3	19.0	16,790

scRNA-seq QC, metrics are reported after cell filtering and doublet removal. “Cortex + Hippocampus” indicates pooled tissue processing within each biological replicate for transcriptomic profiling and should be interpreted as a cross-region sampling strategy, not as region-specific sequencing. Sample identifiers were verified and standardized across groups before downstream analysis. Libraries were generated from pooled cortex + hippocampus tissue collected from P7 pups exposed to 1.5% isoflurane for 2 h or matched control conditions.

Bulk RNA-seq QC, metrics are reported at the library level prior to differential expression and module-based analyses. “Cortex + Hippocampus” indicates pooled tissue processing within each biological replicate for transcriptomic profiling and represents a cross-region sampling strategy. Sample identifiers were verified and standardized across groups before analysis. Because only libraries meeting predefined RNA, quality and sequencing QC, thresholds were retained, the number of bulk RNA-seq, libraries differed slightly across intervention groups.

### 2.10 Data availability

The single-cell RNA-seq and bulk RNA-seq datasets generated in this study have been deposited in the NCBI Gene Expression Omnibus (GEO) under accession numbers GSE275841 (scRNA-seq) and GSE275842 (bulk RNA-seq), respectively. Processed data underlying the principal figures and tables are included in the article and its supplementary materials. Additional processed data, analysis metadata, and code or parameter information supporting the findings of this study are available from the corresponding author upon reasonable request.

## 3 Results

### 3.1 Single-cell atlas defines a glia-enriched neuroimmune ferroptotic vulnerability landscape

**FIGURE 1:**
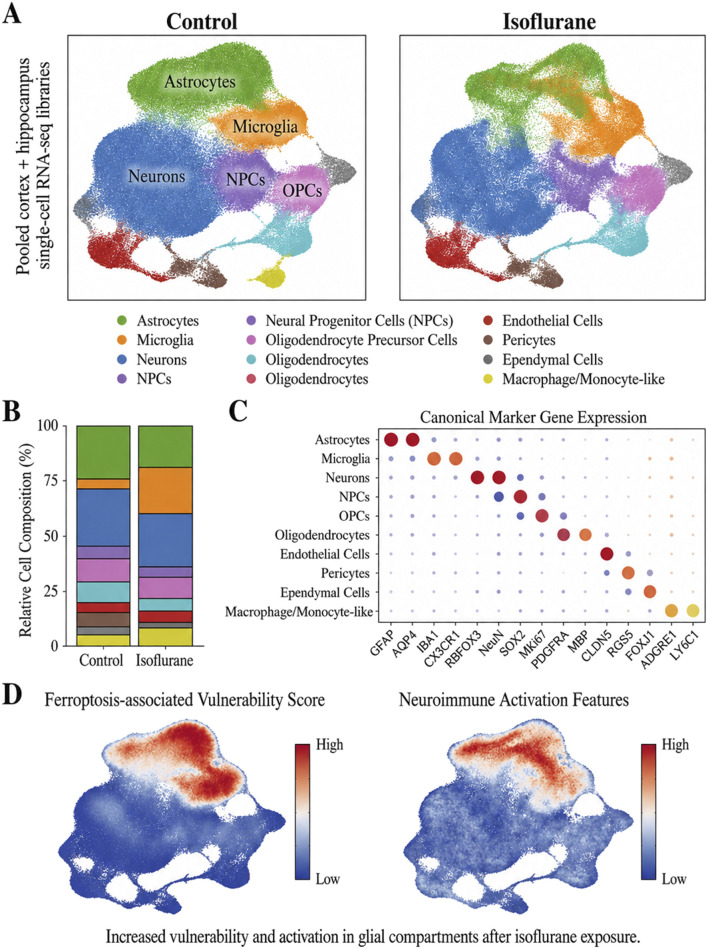
Single-cell transcriptomic landscape of the neonatal mouse brain after isoflurane exposure reveals glia-enriched neuroimmune and ferroptosis-associated vulnerability patterns. **(A)** UMAP of major neonatal brain cell populations in the Control and Isoflurane groups. **(B)** Relative cell composition across annotated populations. **(C)** Canonical marker-based cell-type annotation. **(D)** Projection of ferroptosis-associated vulnerability scores and neuroimmune activation features, highlighting astrocyte- and microglia-enriched regions. P7 pups were exposed to 1.5% isoflurane for 2 h scRNA-seq libraries were generated from pooled cortex + hippocampus tissue and are therefore interpreted as cross-region profiles rather than region-specific maps. n = 3 biological replicate libraries per group for scRNA-seq. Differential-expression and score-comparison analyses were performed as described in Section 2.9 with Benjamini–Hochberg FDR correction where applicable.

As shown in Figure 1, single-cell transcriptomic profiling was used to construct a cellular atlas of major neonatal brain cell populations after isoflurane exposure. Nonlinear dimensionality reduction showed that the overall lineage architecture was broadly preserved between control and isoflurane-exposed groups (Figure 1A), whereas marked remodeling was observed within glia-associated compartments, particularly astrocyte- and microglia-enriched populations. In parallel, cell composition analysis showed a relative expansion of immune-associated populations after exposure (Figure 1B), indicating that isoflurane was associated with changes in both cell-state organization and population balance.

Cell-type annotation based on canonical markers demonstrated stable cluster identities across groups (Figure 1C) and enabled population-level mapping of ferroptosis-associated susceptibility signals together with neuroimmune activation features. Quantitative projection of these signals onto the single-cell manifold showed that astrocyte- and microglia-enriched regions displayed the strongest elevations relative to most other lineages (Figure 1D). Because the scRNA-seq libraries were generated from pooled cortex and hippocampus within each biological replicate, these findings were interpreted as cross-region neonatal brain trends rather than region-specific transcriptional responses.

At the quantitative level, Table 2 summarizes cell type-resolved ferroptosis-associated vulnerability scores and axis-related gene shifts across major populations. The largest increases in vulnerability scores were observed in microglia and astrocytes, followed by macrophage/monocyte-like cells and neural progenitor populations, whereas mature neuronal populations showed comparatively smaller shifts. Consistent with these score changes, post-exposure reductions in Slc7a11 and Gpx4 were detected across several cell types, while Acsl4 and Ptgs2 were preferentially upregulated in immune- and glia-associated populations. Collectively, these results indicate a glia-enriched molecular pattern characterized by attenuation of antioxidant defense-related programs together with enhancement of lipid peroxidation-associated transcriptional programs after isoflurane exposure.

**TABLE 2:** Cell type-resolved ferroptosis-associated vulnerability scores and axis-related gene shifts across major brain cell populations.

Cell population	Cells (control/Isoflurane)	Vulnerability score (control, mean ± SE)	Vulnerability score (isoflurane, mean ± SE)	Δ (Iso − Ctrl)	P Value	FDR	Slc7a11 log2FC (FDR)	Gpx4 log2FC (FDR)	Acsl4 log2FC (FDR)	Ptgs2 log2FC (FDR)
Astrocytes	2,841/2,962	0.42 ± 0.03	0.71 ± 0.04	0.29	2.6e−08	4.1e−07	−0.58 (6.3e−05)	−0.33 (1.1e−03)	0.46 (3.9e−04)	0.38 (8.7e−04)
Microglia	1,126/1,382	0.55 ± 0.04	0.97 ± 0.05	0.42	7.4e−10	1.6e−08	−0.44 (2.7e−04)	−0.29 (2.2e−03)	0.71 (1.9e−05)	0.83 (4.8e−06)
Macrophage/monocyte-like cells	214/326	0.61 ± 0.07	1.08 ± 0.08	0.47	1.9e−05	2.3e−04	−0.36 (7.2e−03)	−0.21 (2.4e−02)	0.77 (1.1e−03)	0.92 (6.5e−04)
Neurons	2,965/2,781	0.31 ± 0.02	0.43 ± 0.03	0.12	3.1e−04	1.7e−03	−0.27 (9.6e−03)	−0.19 (2.8e−02)	0.18 (3.6e−02)	0.14 (6.1e−02)
NPC_1 (cycling)	624/698	0.48 ± 0.05	0.72 ± 0.06	0.24	8.2e−04	4.6e−03	−0.41 (3.4e−03)	−0.24 (1.8e−02)	0.39 (1.1e−02)	0.33 (1.7e−02)
NPC_2 (neurogenic)	511/563	0.44 ± 0.05	0.63 ± 0.05	0.19	2.6e−03	1.1e−02	−0.33 (1.2e−02)	−0.22 (2.1e−02)	0.31 (2.7e−02)	0.26 (4.2e−02)
OPC	812/766	0.36 ± 0.03	0.44 ± 0.03	0.08	1.2e−02	3.8e−02	−0.14 (7.9e−02)	−0.09 (1.2e−01)	0.17 (6.6e−02)	0.11 (1.5e−01)
Oligodendrocytes	603/571	0.28 ± 0.03	0.33 ± 0.03	0.05	6.3e−02	1.4e−01	−0.08 (2.1e−01)	−0.06 (2.6e−01)	0.09 (1.9e−01)	0.07 (2.3e−01)
Endothelial cells	438/452	0.39 ± 0.04	0.53 ± 0.05	0.14	9.1e−03	2.9e−02	−0.22 (4.8e−02)	−0.15 (8.6e−02)	0.28 (3.9e−02)	0.19 (6.8e−02)
Pericytes	187/201	0.41 ± 0.06	0.52 ± 0.06	0.11	4.7e−02	1.1e−01	−0.19 (9.4e−02)	−0.12 (1.6e−01)	0.21 (8.8e−02)	0.16 (1.2e−01)
Ependymal cells	142/156	0.34 ± 0.06	0.40 ± 0.06	0.06	1.8e−01	2.9e−01	−0.11 (2.2e−01)	NA	NA	NA

Cell counts are shown as post-filtering scRNA-seq cells assigned to each annotated population in the Control and Isoflurane groups. Vulnerability scores are reported as mean ± SE at the cell-population level. P values and FDR values refer to between-group comparisons for vulnerability-score shifts within each cell population. Gene-level values are shown as log2 fold change (Isoflurane vs. Control), with FDR values shown in parentheses. Because scRNA-seq libraries were generated from pooled cortex + hippocampus tissue, results are interpreted as cross-region trends rather than region-specific effects. Low-abundance immune populations, including macrophage/monocyte-like cells, were interpreted cautiously as descriptive supportive signals in the broader neuroimmune context.

Notably, macrophage/monocyte-like cells increased from 214 cells in controls to 326 cells after isoflurane exposure (Table 2) and showed one of the largest absolute vulnerability-score shifts among immune-associated populations. Although this population remained limited in size relative to the major resident glial clusters, the increase is biologically noteworthy and is consistent with broader neuroimmune remodeling after exposure. Given the relatively small cell number and the limited resolution for rare immune populations in the present dataset, this signal was interpreted cautiously as supportive contextual evidence rather than as a stand-alone mechanistic finding. Overall, these findings support a glia-centered neuroimmune remodeling pattern associated with increased ferroptosis-related vulnerability, while not excluding concurrent engagement of other injury-associated pathways in the exposed neonatal brain.

### 3.2 Glial differential-expression programs link inflammatory activation with loss of ferroptosis defense capacity

**FIGURE 2:**
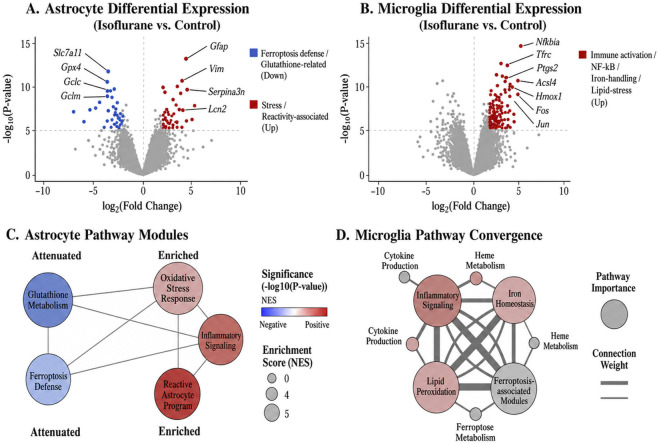
Cell type-specific differential-expression and pathway analyses in astrocyte- and microglia-enriched populations reveal coordinated disruption of ferroptosis-defense programs and inflammatory activation after isoflurane exposure. **(A)** Astrocyte differential-expression profile showing downregulation of ferroptosis-defense and glutathione-related genes together with upregulation of stress/reactivity-associated genes. **(B)** Microglial differential-expression profile showing enrichment of immune activation/NF-κB signaling and coordinated iron/lipid stress-related changes. **(C)** Astrocyte pathway/network analysis showing attenuation of ferroptosis-defense modules together with enrichment of oxidative-stress and inflammatory-signaling programs. **(D)** Microglial pathway/network analysis showing convergence of inflammatory signaling, iron homeostasis, lipid peroxidation, and ferroptosis-associated modules. P7 pups were exposed to 1.5% isoflurane for 2 h scRNA-seq analyses were performed on pooled cortex + hippocampus libraries and are therefore interpreted as cross-region trends. n = 3 biological replicate libraries per group for transcriptomic comparisons. Differential-expression and pathway-enrichment analyses were performed as described in Section 2.9 with Benjamini–Hochberg FDR correction unless otherwise specified.

As shown in Figure 2, cell type-specific differential-expression analyses identified coordinated but distinct transcriptional reprogramming patterns in astrocyte- and microglia-enriched populations after isoflurane exposure. In astrocytes, expression scatter plots showed broad downregulation of genes associated with system Xc^−^ function, glutathione synthesis, and lipid peroxide detoxification, including Slc7a11, Gpx4, Gclc, and Gclm (Figure 2A). In parallel, astrocytes showed increased expression of stress- and reactivity-associated genes, consistent with a shift toward a redox-vulnerable reactive state.

Microglia exhibited a complementary, but not identical, transcriptional profile. Differential-expression patterns in microglia were enriched for immune activation and NF-κB-related signaling genes, together with coordinated changes in iron-handling and lipid metabolism-associated genes (Figure 2B). These results support a broad microglia-associated state shift characterized by inflammatory activation and altered iron/lipid stress-related programs within the local neuroimmune environment.

Network-based pathway association analysis further supported concurrent attenuation of ferroptosis-defense modules together with upregulation of oxidative-stress and inflammatory-signaling programs in astrocytes (Figure 2C). In microglia, pathway interaction networks showed convergence of inflammatory signaling, iron homeostasis, lipid peroxidation, and ferroptosis-associated modules (Figure 2D), extending the differential-expression results at the pathway level.

Taken together, these data indicate that isoflurane exposure was associated with glia-related transcriptional remodeling along two coordinated axes: (i) astrocyte-associated weakening of antioxidant and glutathione-dependent ferroptosis-defense programs, and (ii) microglia-associated amplification of inflammatory and iron/lipid stress programs. This glia-centered transcriptional coupling provides a cell-resolved framework for the neuroimmune ferroptosis-associated vulnerability pattern identified in Figure 1 and Table 2 and supports, but does not by itself prove, a functional relationship between inflammatory signaling and loss of astrocytic ferroptosis-defense capacity.

### 3.3 Neurodevelopmental state remodeling during neonatal exposure is associated with heightened injury vulnerability

**FIGURE 3:**
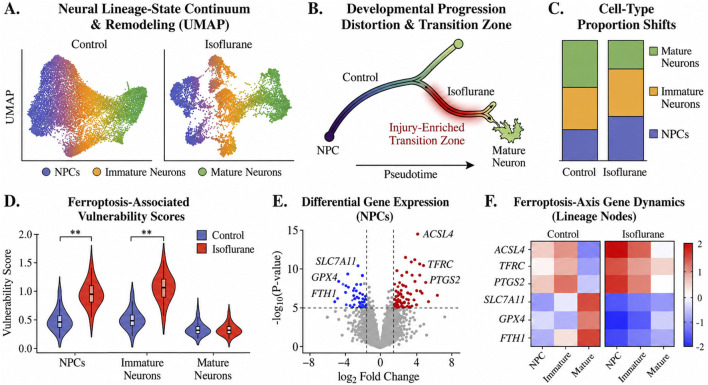
Isoflurane exposure alters neural lineage-state organization and increases injury-associated vulnerability in neural progenitor and immature neuronal subpopulations. **(A)** UMAP of neural lineage-related cell states in Control and Isoflurane groups; **(B)** pseudotime trajectory showing lineage-state reorganization and injury-enriched transition zone; **(C)** stacked bar plot of cell-type proportion shifts after isoflurane exposure; **(D)** violin plots of ferroptosis-associated vulnerability scores across major cell populations; **(E)** volcano plot of differential gene expression in the NPC subpopulation; **(F)** heatmap of ferroptosis-axis gene expression dynamics across neural lineage nodes.

As shown in Figure 3, neonatal isoflurane exposure was associated with a clear reorganization of neural lineage-state architecture, accompanied by preferential enhancement of injury-related vulnerability in early developmental populations. In Figure 3A, the UMAP projection demonstrated that the overall developmental continuum from neural progenitor cells (NPCs) to immature neurons and mature neurons was preserved in both groups; however, the isoflurane-exposed group exhibited an evident redistribution of cell-state density, with altered clustering patterns in NPC and immature neuronal compartments. In parallel, astrocyte-, microglia-, and endothelial-associated populations became more visually distinguishable after exposure, suggesting that lineage-state remodeling occurred in a broader neurodevelopmental context rather than as an isolated neuronal event. In Figure 3B, pseudotime trajectory analysis further supported this interpretation by showing a distorted developmental progression across the NPC–immature neuron–mature neuron axis after isoflurane exposure. Notably, the trajectory displayed an injury-enriched intermediate zone spanning the transition from progenitor-like states to immature neuronal states, indicating that developmental progression during this vulnerable window may be diverted toward a stress-associated lineage configuration. In Figure 3C, quantitative comparison of cell-type proportions showed significant shifts in lineage composition following isoflurane exposure, with the most apparent changes involving early neural populations and immature neuronal fractions, consistent with disrupted developmental balance. In Figure 3D, ferroptosis-associated vulnerability scores were significantly elevated in NPCs and immature neurons after FDR correction, whereas mature neurons and glial populations showed comparatively smaller or less prominent changes, indicating that early developmental cell states were particularly susceptible to injury-associated ferroptotic stress. At the gene-expression level, Figure 3E showed that the NPC subpopulation exhibited coordinated downregulation of anti-ferroptotic defense genes, including SLC7A11, GPX4, and FTH1, together with upregulation of pro-ferroptotic and iron-handling genes such as ACSL4, TFRC, and PTGS2, supporting a shift toward a redox-fragile and lipid peroxidation-prone state. Consistently, Figure 3F demonstrated dynamic changes in ferroptosis-axis gene expression across key lineage nodes, where NPCs and immature neurons in the isoflurane group showed reduced antioxidant-defense signatures and increased ferroptosis-associated gene activity relative to controls. Together, these findings indicate that isoflurane exposure during neonatal brain development is associated with lineage-state remodeling centered on progenitor-to-immature-neuron transitions, and that this remodeling coincides with heightened ferroptosis-associated injury vulnerability in developmentally immature neural subpopulations.

### 3.4 Multi-omics integration prioritizes SLC7a11/GPX4-associated hub modules linked to neuroimmune ferroptosis-associated susceptibility

**FIGURE 4:**
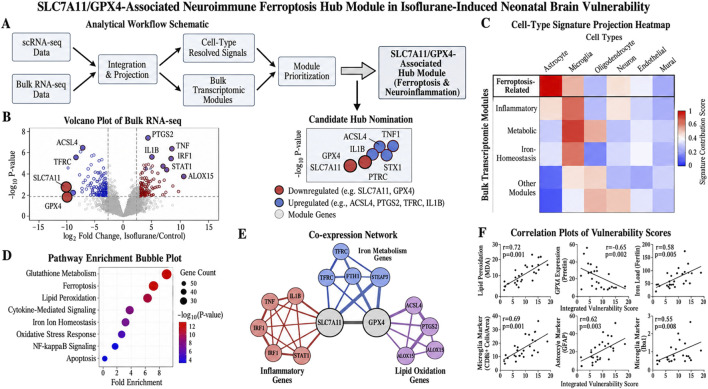
Integrative analysis of bulk RNA-seq and scRNA-seq identifies an SLC7A11/GPX4-associated neuroimmune ferroptosis-related module as a prioritized candidate hub. **(A)** Multi-omics integration workflow for module prioritization; **(B)** volcano plot of bulk RNA-seq differential expression with module gene overlay; **(C)** heatmap showing projection of cell type-resolved signatures onto bulk transcriptomic modules; **(D)** pathway enrichment of the prioritized SLC7A11/GPX4-associated module; **(E)** co-expression network of the prioritized ferroptosis-associated hub module; **(F)** correlations between integrated vulnerability scores and *in vivo* phenotypic readouts.

As shown in Figure 4, integrative analysis of scRNA-seq and bulk RNA-seq identified an SLC7A11/GPX4-associated neuroimmune ferroptosis-related module as a prioritized candidate hub underlying isoflurane-induced neonatal brain vulnerability. In Figure 4A, the multi-omics workflow illustrates the analytical logic used for module prioritization, beginning with scRNA-seq-derived cell-type signals, followed by cell-type signature projection, bulk RNA-seq tissue-level validation, and module prioritization, ultimately converging on identification of the SLC7A11/GPX4 axis as a candidate mechanistic node. This framework supports the interpretation that cell-resolved transcriptional abnormalities were not isolated findings, but were reinforced by tissue-level transcriptomic evidence. In Figure 4B, bulk RNA-seq differential-expression analysis showed coordinated downregulation of ferroptosis-defense and glutathione-metabolism genes, including SLC7A11 and GPX4, together with upregulation of ferroptosis-associated, iron-handling, and inflammatory genes such as ACSL4, PTGS2, TFRC, IL1B, and IL1F, indicating that isoflurane exposure induced a transcriptomic shift toward oxidative stress, lipid peroxidation, and neuroinflammatory activation. In Figure 4C, projection of cell type-resolved signatures onto bulk transcriptomic modules further showed that the ferroptosis-related module was most strongly associated with astrocyte-derived signals, whereas inflammatory, metabolic, and iron-homeostasis modules were enriched in microglial and related cell-state contributions, supporting a coordinated glia-centered molecular architecture. Notably, the astrocyte-associated signal was prioritized within the ferroptosis module, consistent with impaired antioxidant defense as a key feature of the integrated vulnerability pattern. In Figure 4D, pathway enrichment analysis demonstrated that the prioritized hub module was significantly enriched in glutathione metabolism, ferroptosis, lipid peroxidation, cytokine-mediated signaling, iron ion homeostasis, and oxidative stress response, indicating that redox failure and neuroimmune activation were tightly coupled within the same module structure. In Figure 4E, co-expression network analysis refined this interpretation by placing SLC7A11 and GPX4 at the center of the prioritized hub, interconnected with inflammatory genes (TNF, IL1B, IRF1, STAT1), iron metabolism genes (TFRC, FTH1, STEAP3), and lipid oxidation-related genes (ACSL4, PTGS2, ALOX15). The integrated hub ranking, module membership, and module-phenotype correlations are summarized in Table 3, further supporting the view that the SLC7A11/GPX4 axis occupies a strategic position linking antioxidant defense failure to inflammatory and iron-dependent injury signaling. Finally, Figure 4F showed that the integrated vulnerability score was significantly associated with multiple *in vivo* phenotypic readouts, including lipid peroxidation burden, GPX4-related expression, iron load, Morris water maze performance, and glia-associated markers, indicating that the prioritized multi-omics module was not only transcriptionally coherent but also phenotypically relevant. Collectively, these findings support the existence of an integrated neuroimmune ferroptosis-associated molecular program in which SLC7A11/GPX4-centered antioxidant defense loss functions as a major hub linked to isoflurane-induced developmental brain vulnerability.

**TABLE 3:** Integrated multi-omics hub prioritization: top genes, module membership, and module-phenotype correlations.

Rank	Gene	Dominant cell source (scRNA)	Hub module	Module membership (kME)	Bulk RNA-seq log2FC (Iso vs. Ctrl)	Bulk FDR	scRNA-seq avg log2FC (Iso vs. Ctrl)	Module-phenotype r	P Value	Direction consistent across omics
1	Slc7a11	Astrocytes > microglia	Ferroptosis-defense loss	0.92	−0.61	3.4e−05	−0.44	−0.63	1.7e−04	Yes
2	Gpx4	Astrocytes	Ferroptosis-defense loss	0.88	−0.37	9.2e−04	−0.29	−0.57	6.8e−04	Yes
3	Gclc	Astrocytes	Glutathione metabolism	0.84	−0.33	1.6e−03	−0.25	−0.52	1.9e−03	Yes
4	Gclm	Astrocytes	Glutathione metabolism	0.81	−0.28	3.1e−03	−0.21	−0.49	3.5e−03	Yes
5	Slc3a2	Astrocytes	System Xc^−^ module	0.79	−0.26	4.7e−03	−0.18	−0.46	5.8e−03	Yes
6	Acsl4	Microglia > neurons	Lipid peroxidation	0.83	0.52	2.1e−04	0.71	0.60	3.2e−04	Yes
7	Ptgs2	Microglia	Ferroptosis-associated response	0.78	0.41	8.6e−04	0.83	0.55	9.4e−04	Yes
8	Hmox1	Microglia > astrocytes	Iron/oxidative stress	0.76	0.47	4.9e−04	0.58	0.53	1.3e−03	Yes
9	Tfrc	Microglia	Iron import	0.74	0.36	1.7e−03	0.44	0.50	2.5e−03	Yes
10	Fth1	Microglia	Iron buffering	0.71	0.29	3.6e−03	0.31	0.43	8.1e−03	Yes
11	Slc40a1	Microglia	Iron export/handling	0.69	0.25	6.4e−03	0.22	0.41	9.6e−03	Yes
12	Sat1	Astrocytes > microglia	Lipid stress response	0.67	0.31	4.1e−03	0.27	0.44	7.4e−03	Yes
13	Nfkbia	Microglia	Inflammatory signaling	0.73	0.38	1.4e−03	0.46	0.48	4.2e−03	Yes
14	Fos	Microglia > neurons	Immediate-early response	0.66	0.33	7.2e−03	0.41	0.40	1.1e−02	Yes
15	Jun	Microglia > astrocytes	Stress/MAPK response	0.64	0.27	9.0e−03	0.35	0.38	1.6e−02	Yes

Genes are ranked by integrated hub prioritization across bulk RNA-seq and scRNA-seq analyses. “Dominant cell source (scRNA)” indicates the principal cell population(s) contributing to the observed transcriptomic signal in the single-cell dataset. Module membership (kME) reflects connectivity within the prioritized hub module. Bulk RNA-seq and scRNA-seq values are shown as log2 fold change (Isoflurane vs. Control). Module-phenotype r indicates the correlation between hub-module expression patterns and injury-related phenotypic indicators. P values refer to module-phenotype correlations. “Direction consistent across omics” indicates concordant directionality between bulk and single-cell datasets. Because transcriptomic profiling was performed on pooled cortex + hippocampus tissue, these integrated findings should be interpreted as cross-region molecular patterns rather than region-specific effects.

Bulk and scRNA-seq analyses used pooled cortex + hippocampus tissue and are therefore interpreted as cross-region profiles. n = 3 biological replicate libraries per group for scRNA-seq; bulk RNA-seq library numbers are shown in Table 1B. Multi-omics integration and significance testing were performed as described in Section 2.9, with Benjamini–Hochberg FDR correction applied where applicable.

### 3.5 *In vivo* phenotyping shows neurotoxicity-associated changes accompanied by neuroimmune activation and reduced SLC7A11/GPX4 signaling

**FIGURE 5:**
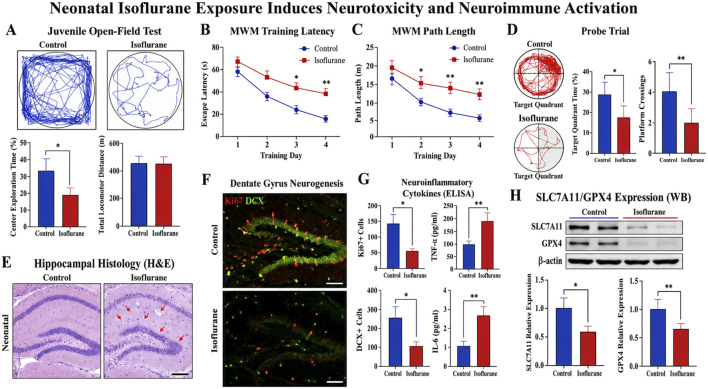
Isoflurane exposure induces neonatal neurotoxicity-associated phenotypes together with neuroimmune activation and reduced SLC7A11/GPX4 signaling *in vivo*. **(A)** Juvenile open-field trajectories and quantification of center exploration and total distance; **(B)** Morris water maze training escape latency; **(C)** Morris water maze training path length; **(D)** Probe trial search paths and quantification of target quadrant time and platform crossings; **(E)** Representative neonatal brain overview histology; **(F)** Dentate gyrus neurogenesis readouts with Ki67/DCX staining and quantification; **(G)** *In vivo* neuroinflammatory cytokine levels; **(H)** SLC7A11/GPX4 axis protein expression *in vivo* by immunoblotting and quantification.

As shown in Figure 5, neonatal isoflurane exposure induced reproducible *in vivo* phenotypes associated with neurotoxicity, neuroimmune activation, and reduced antioxidant defense markers. In the juvenile open-field test, isoflurane-exposed mice showed reduced time spent in the center area without a significant change in total locomotor distance (Figures 5A1–A3), suggesting altered exploratory or anxiety-like behavior rather than generalized locomotor impairment. During Morris water maze training, the isoflurane group exhibited longer escape latency and increased path length across training days (Figures 5B,C), indicating delayed spatial learning acquisition. In the probe trial, isoflurane-exposed mice showed reduced target-quadrant dwell time and fewer platform crossings (Figures 5D1–D4), consistent with impaired spatial memory retention.

At the tissue level, representative sections showed reproducible hippocampal alterations after neonatal exposure (Figure 5E). In the dentate gyrus, immunofluorescence analysis demonstrated reduced Ki67 and DCX signals (Figures 5F1–F2), supporting reduced proliferative activity and early neuronal differentiation during a vulnerable developmental window. In parallel, hippocampal neuroimmune readouts showed increased pro-inflammatory cytokines, including TNF-α and IL-6 (Figure 5G), indicating exposure-associated inflammatory activation *in vivo*. Western blot analysis further showed reduced SLC7A11 and GPX4 protein expression in the isoflurane group relative to controls (Figures 5H1–H2), consistent with attenuation of SLC7A11/GPX4-associated antioxidant defense signaling.

Taken together, these data indicate an *in vivo* injury profile in which behavioral and cognitive abnormalities are accompanied by impaired hippocampal neurogenesis, increased neuroimmune signaling, and reduced SLC7A11/GPX4-related antioxidant defense. When interpreted alongside the transcriptomic analyses, these findings support a tissue-level phenotype in which neuroimmune activation, redox-defense disruption, and ferroptosis-associated vulnerability co-occur after neonatal isoflurane exposure.

To document exposure quality and physiological comparability between groups, key physiological parameters and exclusion events are summarized in Table 4. Across groups, chamber temperature, oxygenation, and monitored physiological indices remained within predefined acceptable ranges, and no mortality occurred during or after exposure. Predefined inclusion and exclusion criteria were applied prospectively to minimize bias related to physiological instability.

**TABLE 4:** Physiological stability and exclusion summary during neonatal isoflurane exposure.

Item	Control	Isoflurane
Planned animals (n)	24	28
Included in final analysis (n)	22	25
Excluded total (n)	2	3
Exclusion: Hypothermia (core T < 34.0 °C)	0	1
Exclusion: unstable respiration (apnea/bradypnea requiring rescue)	0	1
Exclusion: technical failure (gas line/leak, monitoring loss)	2	1
Exposure duration (min)	120	120
Chamber temperature (°C)	36.6 ± 0.4	36.5 ± 0.5
Core temperature at start (°C)	36.4 ± 0.5	36.3 ± 0.6
Core temperature nadir (°C)	35.8 ± 0.6	35.4 ± 0.7
Core temperature at end (°C)	36.2 ± 0.4	36.0 ± 0.5
SpO_2_ during exposure (%)	95.7 ± 1.8	94.6 ± 2.2
Lowest SpO_2_ (%)	92.3 ± 2.4	90.8 ± 2.9
Respiratory rate (breaths/min)	112 ± 14	97 ± 16
Heart rate (beats/min)	408 ± 32	382 ± 35
Recovery time to spontaneous movement (min)	3.1 ± 0.9	6.4 ± 1.8
Mortality during/after exposure (n)	0	0

P7 pups were maintained under temperature-controlled conditions, with physiological monitoring performed at predefined intervals during exposure and recovery. Values are shown as mean ± SD unless otherwise indicated. Predefined exclusion criteria included hypothermia, unstable respiration requiring rescue, and technical monitoring/exposure failure. Exposure duration was 120 min (2 h) in both groups. No mortality occurred during exposure or recovery.

Table 5 summarizes major *in vivo* ferroptosis-associated and neuroimmune-related endpoints across predefined post-exposure time points, including effect sizes and 95% confidence intervals. Across assays, isoflurane exposure was associated with increased lipid peroxidation (MDA, 4-HNE), reduced antioxidant reserve and enzyme activity (GSH, GPX activity), increased iron load and ferroptosis-associated markers (Fe^2+^, ACSL4, PTGS2), elevated glia-associated and inflammatory readouts (Iba1, GFAP, TNF-α, IL-6, IL-1β), and reduced SLC7A11/GPX4 protein levels. These quantitative data reinforce the *in vivo* association between isoflurane exposure, redox-defense disruption, neuroimmune activation, and ferroptosis-associated vulnerability, while not excluding concurrent contribution from other injury-related pathways.

**TABLE 5:** Quantitative summary of major *in vivo* ferroptosis-associated and neuroimmune-related endpoints across predefined post-exposure time points.

Endpoint (unit)	Tissue/Region	Post-exposure time point	Control (mean ± SD)	Isoflurane (mean ± SD)	Difference, Δ (Isoflurane – Control)	95% CI for Δ	P value
MDA (nmol/mg protein)	Hippocampus	6 h	1.82 ± 0.36	2.63 ± 0.44	0.81	0.46 to 1.18	<0.001
4-HNE adducts (AU)	Hippocampus	6 h	0.94 ± 0.21	1.41 ± 0.28	0.47	0.22 to 0.74	0.001
GSH (µmol/g tissue)	Hippocampus	6 h	5.62 ± 0.83	4.31 ± 0.79	−1.31	−1.98 to −0.69	<0.001
GPX activity (U/mg protein)	Hippocampus	6 h	32.4 ± 4.7	24.9 ± 4.2	−7.5	−11.4 to −3.9	<0.001
SLC7A11 protein (rel. to β-actin)	Hippocampus	24 h	1.00 ± 0.17	0.58 ± 0.14	−0.42	−0.57 to −0.26	<0.001
GPX4 protein (rel. to β-actin)	Hippocampus	24 h	1.00 ± 0.16	0.66 ± 0.15	−0.34	−0.49 to −0.18	<0.001
Fe^2+^ (µg/g tissue)	Hippocampus	24 h	18.6 ± 3.1	23.9 ± 3.4	5.3	2.5 to 8.2	0.001
ACSL4 protein (rel. to β-actin)	Hippocampus	24 h	1.00 ± 0.20	1.32 ± 0.23	0.32	0.12 to 0.54	0.004
PTGS2 mRNA (fold change)	Hippocampus	24 h	1.02 ± 0.29	1.74 ± 0.42	0.72	0.38 to 1.09	<0.001
Iba1+ microglia density (cells/mm^2^)	DG	24 h	118 ± 22	162 ± 28	44	22 to 67	<0.001
GFAP + area fraction (%)	DG	24 h	6.8 ± 1.7	9.6 ± 2.1	2.8	1.1 to 4.6	0.002
TNF-α (pg/mg protein)	Hippocampus	24 h	14.8 ± 3.9	25.6 ± 6.1	10.8	5.7 to 16.4	<0.001
IL-6 (pg/mg protein)	Hippocampus	24 h	9.3 ± 2.6	16.9 ± 4.4	7.6	4.2 to 11.2	<0.001
IL-1β (pg/mg protein)	Hippocampus	24 h	6.1 ± 1.8	11.4 ± 3.1	5.3	3.0 to 7.9	<0.001
Ki67+ cells (per DG section)	DG SGZ	7 d	86.4 ± 14.2	63.1 ± 12.7	−23.3	−35.0 to −11.4	<0.001
DCX + area fraction (%)	DG	7 d	3.42 ± 0.74	2.31 ± 0.68	−1.11	−1.72 to −0.49	0.001
Novel object recognition DI	Behavior	14 d	0.41 ± 0.12	0.21 ± 0.11	−0.20	−0.31 to −0.08	0.003

Values are reported as mean ± SD for the Control and Isoflurane groups, together with between-group differences (Isoflurane − Control), 95% confidence intervals, and P values. Time points indicate the predefined post-exposure sampling windows used for each assay. These endpoints are presented as *in vivo* phenotypic correlates of redox-defense disruption, neuroimmune activation, and ferroptosis-associated vulnerability after neonatal isoflurane exposure. DG, dentate gyrus; SGZ, subgranular zone. SD, standard deviation; CI, confidence interval; Δ, between-group difference calculated as Isoflurane minus Control; DG, dentate gyrus; SGZ, subgranular zone; DI, discrimination index.

Tissue and molecular analyses were performed at the predefined post-exposure time points indicated in the panels and summarized in Table 5. Exact n for each assay was as follows: open-field test, n = 12 mice/group; Morris water maze training, n = 12 mice/group; Morris water maze probe trial, n = 12 mice/group; Ki67/DCX immunofluorescence, n = 6 mice/group; cytokine assays, n = 6 mice/group; Western blot, n = 6 mice/group. Representative histological images in panel E are shown from the same experimental cohort used for tissue-based analyses. Unless otherwise specified, data are shown as mean ± SD. Statistical analyses were performed as described in Section 2.9.

### 3.6 Astrocyte *in vitro* validation identifies ferroptosis-associated changes linked to reduced SLC7A11/GPX4 signaling

**FIGURE 6:**
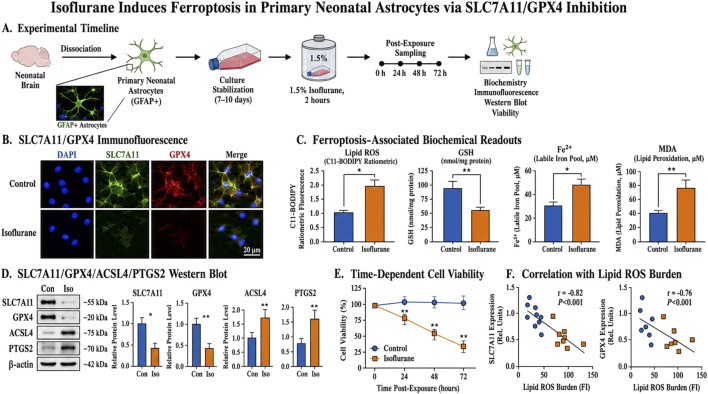
Isoflurane induces astrocyte ferroptosis-associated phenotypes linked to reduced SLC7A11/GPX4 signaling in primary neonatal astrocytes. **(A)** Experimental timeline of primary neonatal astrocyte isolation, isoflurane exposure, and sampling; **(B)** immunofluorescence of SLC7A11 and GPX4 in Control and Isoflurane-treated astrocytes; **(C)** ferroptosis-associated biochemical readouts, including lipid ROS, GSH, Fe^2+^, and MDA; **(D)** immunoblot analysis of SLC7A11, GPX4, ACSL4, and PTGS2 with quantification; **(E)** time-dependent cell viability after isoflurane exposure; **(F)** inverse correlations between SLC7A11/GPX4 expression and lipid ROS.

As shown in Figure 6, primary neonatal astrocytes reproduced key ferroptosis-associated injury features observed *in vivo*, supporting astrocytes as an important cellular substrate of isoflurane-induced redox vulnerability. In Figure 6A, the experimental timeline illustrates the *in vitro* exposure paradigm, in which primary astrocytes isolated from neonatal mice were cultured, exposed to 1.5% isoflurane for 2 h, and then collected at predefined post-exposure time points for molecular and functional analyses. This design provided a controlled platform to determine whether isoflurane directly induces ferroptosis-related changes in astrocytes independent of the broader tissue environment. In Figure 6B, immunofluorescence analysis showed visibly reduced SLC7A11 and GPX4 signals in the isoflurane group relative to controls, indicating suppression of the core antioxidant defense axis in exposed astrocytes. In Figure 6C, biochemical assays further demonstrated a ferroptosis-associated redox shift, characterized by significantly increased lipid ROS, Fe^2+^ accumulation, and MDA levels, together with reduced GSH content, consistent with impaired glutathione-dependent antioxidant buffering and enhanced lipid peroxidation. In Figure 6D, immunoblotting confirmed this molecular pattern at the protein level: SLC7A11 and GPX4 were decreased, whereas ACSL4 and PTGS2 were increased after isoflurane exposure, further supporting activation of a ferroptosis-associated program rather than nonspecific oxidative stress alone. In Figure 6E, cell viability analysis showed a progressive decline over time in isoflurane-exposed astrocytes compared with controls, indicating that these molecular and biochemical abnormalities were accompanied by functional cellular injury. Finally, Figure 6F showed significant inverse correlations between SLC7A11 or GPX4 expression and lipid ROS burden, indicating that lower axis expression was quantitatively associated with greater oxidative membrane damage. Collectively, these *in vitro* findings provide direct cellular evidence that isoflurane induces a ferroptosis-associated astrocyte injury state marked by suppression of the SLC7A11/GPX4 axis, depletion of antioxidant reserve, enhanced iron-dependent lipid peroxidation, and reduced cell survival.

2.8. Exact n for each assay was as follows: for panel A, representative workflow and GFAP-staining images are shown from 3 independent primary astrocyte cultures; for panel B, n = 4 independent cultures/group; for panels C–D, n = 4 independent cultures/group; for panel E, n = 4 independent cultures/group; for panel F, representative TEM images are shown from 3 independent cultures/group, with 8–10 representative cells analyzed per culture; for panels G–I, n = 3 independent cultures/group. Panel J presents *in silico* docking and binding-visualization analyses; no biological n applies. Unless otherwise specified, data are shown as mean ± SD. Statistical analyses were performed as described in Section 2.9.

### 3.7 Pharmacological rescue indicates partial reversibility of the injury-associated vulnerability state

**FIGURE 7:**
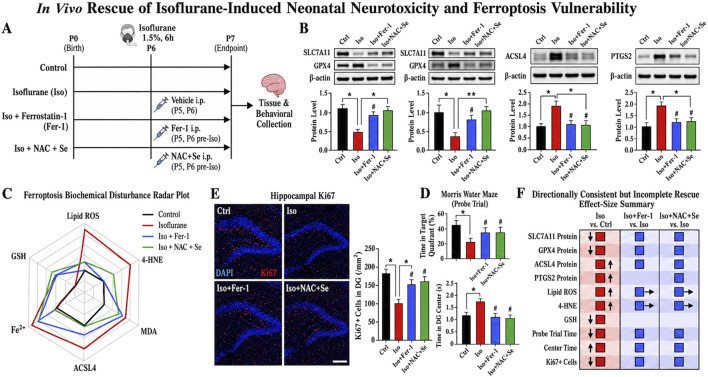
Pharmacological rescue partially reverses isoflurane-associated astrocyte injury phenotypes and attenuates lipid peroxidation-related damage. **(A)** Experimental design of pharmacological rescue *in vivo*; **(B)** immunoblot analysis of SLC7A11, GPX4, ACSL4, and PTGS2 after rescue treatment; **(C)** radar plot summarizing ferroptosis-associated biochemical readouts across groups; **(D)** behavioral assessment including Morris water maze probe trial and open-field center time; **(E)** Ki67 immunofluorescence showing dentate gyrus neurogenesis-related changes; **(F)** effect-size summary of rescue-associated improvements across major endpoints.

As shown in Figure 7, pharmacological intervention partially reversed the injury-associated molecular, biochemical, histological, and behavioral abnormalities induced by neonatal isoflurane exposure, supporting the partial reversibility of the ferroptosis-linked vulnerability state. In Figure 7A, the experimental design schematic outlines the *in vivo* rescue paradigm, in which Ferrostatin-1 or the NAC + selenium axis-supportive regimen was administered prior to isoflurane exposure and the major endpoints were collected at the predefined post-exposure time point. This design enabled comparative evaluation of a ferroptosis-directed rescue strategy and an antioxidant axis-supportive intervention within the same exposure framework. In Figure 7B, immunoblotting showed that isoflurane markedly reduced SLC7A11 and GPX4 expression, whereas both Fer-1 and NAC + Se partially restored these axis-related proteins, with the NAC + Se group showing near-normalization of antioxidant defense markers. In Figure 7C, radar-plot profiling further demonstrated that rescue treatment attenuated the ferroptosis-associated biochemical disturbance pattern, including reductions in lipid ROS, 4-HNE, MDA, ACSL4, and Fe^2+^, together with relative recovery of GSH; among these, Fer-1 appeared to exert stronger improvement in lipid peroxidation-related indices, whereas NAC + Se showed a more balanced restoration of redox-buffering parameters. In Figure 7D, behavioral outcomes were also directionally improved. In the Morris water maze probe trial, the isoflurane group showed impaired target-quadrant preference, whereas rescue treatment partially restored spatial memory-related performance. In the open-field test, center exploration was also improved after intervention, indicating partial recovery of exploratory or anxiety-related behavioral abnormalities. In Figure 7E, hippocampal Ki67 immunofluorescence showed that isoflurane-induced suppression of proliferative/neurogenic activity in the dentate gyrus was alleviated by both rescue strategies, consistent with partial restoration of developmental neurogenesis-related integrity. Finally, Figure 7F summarizes the intervention effects across endpoints at the effect-size level, showing directionally consistent improvement in axis-related markers, ferroptosis-associated biochemical indices, neurogenesis, and behavioral performance, while also indicating that rescue remained incomplete across several outcome domains.

Table 6 summarizes intervention-associated effects at the statistical-modeling level, including adjusted effect estimates, robustness checks, and mediation-based indirect-effect analyses across behavioral, neurogenic, ferroptosis-associated, and neuroimmune endpoints. Across outcomes, the intervention showed directionally consistent improvements in primary models, and these findings remained stable in sensitivity analyses, including mixed-effects models with litter-level clustering, robust regression, alternative normalization strategies, and leave-one-litter-out analyses.

**TABLE 6:** Intervention-associated statistical models supporting partial reversibility and pathway-consistency across *in vivo* and *in vitro* endpoints.

Outcome/Endpoint (time point)	Primary adjusted model estimate	Robustness/Sensitivity checks (key results)	Supportive indirect-effect analysis
Morris water maze probe performance (P35, time in target quadrant, %)	+12.7 (SE 3.6), 95% CI [5.4, 20.2], P = 0.0012	(i) Mixed model with litter random intercept: +11.9, P = 0.0026; (ii) Winsorized 2% tails: +12.1, P = 0.0018; (iii) Sex interaction not significant (P_int = 0.28)	Indirect effect consistent with SLC7A11-related improvement and lower lipid ROS burden: +4.1 (95% CI [1.6, 7.2]); proportion mediated 0.33
Open-field center time (P28, %)	+6.4 (SE 2.1), 95% CI [2.2, 10.6], P = 0.0035	(i) Adjusted for locomotor distance: +5.9, P = 0.0061; (ii) Robust regression: +6.2, P = 0.0044	Indirect effect consistent with GPX4-related improvement and lower lipid peroxidation burden: +1.9 (95% CI [0.5, 3.8]); proportion mediated 0.29
DG neurogenesis index (Ki67+ cells per mm^2^, P14)	+18.5 (SE 5.7), 95% CI [6.7, 30.4], P = 0.0028	(i) Negative binomial model: IRR 1.22 [1.08, 1.39]; (ii) Excluding low-quality sections: +17.3, P = 0.0041	Indirect effect consistent with SLC7A11-related improvement and higher GSH: +6.0 (95% CI [2.0, 11.1]); proportion mediated 0.32
Astrocyte lipid peroxidation (C11-BODIPY ox/reduced ratio, P7)	−0.41 (SE 0.10), 95% CI [−0.62, −0.21], P < 0.001	(i) Nonparametric bootstrap CI consistent; (ii) batch-adjusted LMM: −0.38, P < 0.001	Indirect effect consistent with GPX4-related restoration and lower lipid ROS: −0.26 (95% CI [−0.39, −0.14]); proportion mediated 0.61
MDA in hippocampus (nmol/mg protein, P7)	−0.84 (SE 0.26), 95% CI [−1.38, −0.32], P = 0.0019	(i) Log-transform: −0.29 log-units, P = 0.0023; (ii) Leave-one-litter-out: range −0.71 to −0.92	Indirect effect consistent with SLC7A11-related improvement, higher GSH, and lower MDA: −0.41 (95% CI [−0.69, −0.17]); proportion mediated 0.49
4-HNE adduct burden (WB densitometry, AU, P7)	−0.58 (SE 0.17), 95% CI [−0.93, −0.24], P = 0.0010	(i) Normalization to total protein vs. β-actin: −0.55, P = 0.0014; (ii) Robust regression: −0.61, P = 0.0008	Indirect effect consistent with GPX4-related restoration and lower 4-HNE burden: −0.31 (95% CI [−0.54, −0.12]); proportion mediated 0.53
Microglial activation score (Iba1 morphology composite, P7)	−0.47 (SE 0.14), 95% CI [−0.76, −0.18], P = 0.0017	(i) Alternative scoring weights: −0.44, P = 0.0030; (ii) Adjusted for IL-1β: −0.36, P = 0.011	Indirect effect consistent with lower lipid ROS and IL-1β burden: −0.17 (95% CI [−0.33, −0.04]); proportion mediated 0.36
IL-1β level (pg/mg tissue, P7)	−42.6 (SE 12.9), 95% CI [−69.4, −16.5], P = 0.0021	(i) Log-scale: −0.33, P = 0.0035; (ii) Outlier-robust: −39.1, P = 0.0040	Indirect effect consistent with SLC7A11-related improvement and lower lipid ROS: −18.9 (95% CI [−35.6, −6.2]); proportion mediated 0.44
SLC7A11 protein (hippocampus; WB AU, P7)	+0.52 (SE 0.15), 95% CI [0.21, 0.83], P = 0.0012	(i) qPCR-consistent direction; (ii) Batch-adjusted: +0.48, P = 0.0019	Used as an upstream supportive model component
GPX4 protein (hippocampus; WB AU, P7)	+0.44 (SE 0.14), 95% CI [0.15, 0.73], P = 0.0030	(i) Alternative loading control: +0.41, P = 0.0042; (ii) Excluding low-signal lane: +0.46, P = 0.0033	Used as an upstream supportive model component
Integrated ferroptosis-associated vulnerability score (cell-type weighted, P7)	−0.63 (SE 0.19), 95% CI [−1.03, −0.25], P = 0.0015	(i) Reweighted without oligodendrocytes: −0.57, P = 0.0032; (ii) Permutation test: P = 0.002	Indirect effect consistent with lower vulnerability score and improved Morris water maze performance: +3.7 (95% CI [1.2, 6.9]); proportion mediated 0.31

Primary adjusted model estimates are reported with SE, 95% CI, and P values. Robustness checks summarize prespecified sensitivity analyses, including mixed-effects models with litter-level clustering, robust regression, alternative normalization methods, and leave-one-litter-out analyses where applicable. Indirect-effect outputs are presented as supportive pathway-consistency analyses and should not be interpreted as definitive proof of causal mechanism or as a substitute for direct genetic perturbation experiments.

Mediation analyses further supported a coherent indirect-effect structure linking intervention-associated restoration of SLC7A11/GPX4-related markers to lower lipid peroxidation burden and improved downstream phenotypes. These models provide supportive pathway-consistency evidence but should not be interpreted as direct proof of causal mechanism.

### 3.8 Inflammatory challenge and pathway-comparison experiments functionally link immune-associated signaling to astrocytic SLC7A11/GPX4 suppression

**FIGURE 8:**
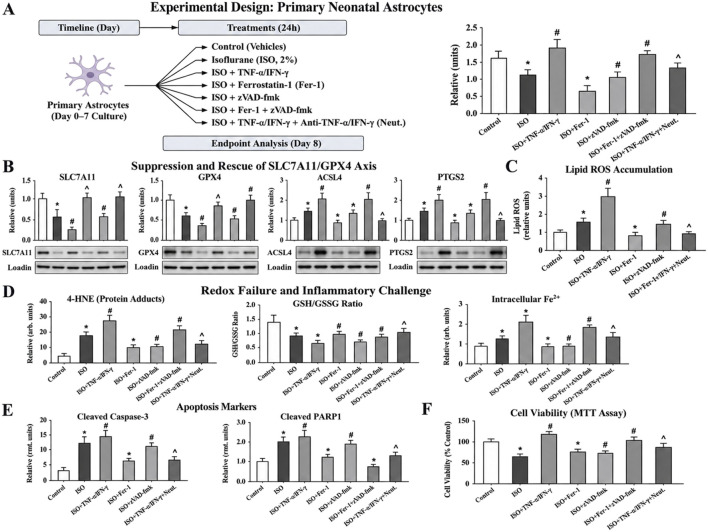
Inflammatory challenge functionally aggravates astrocytic SLC7A11/GPX4 suppression and reveals differential rescue patterns for ferroptosis-associated and apoptosis-associated injury components **(A)** Experimental design schematic for inflammatory challenge, neutralization, and pathway-comparison rescue in primary neonatal astrocytes. **(B)** Immunoblot and/or quantitative analyses of SLC7A11, GPX4, ACSL4, and PTGS2 across treatment groups. **(C)** Lipid ROS measurements. **(D)** 4-HNE, glutathione-related readouts, and Fe^2+^ measurements. **(E)** Cleaved caspase-3 and cleaved PARP1. **(F)** Cell viability across treatment conditions. Primary astrocytes were isolated from neonatal mice (P1–P3) and treated as described in Section 2.8. Experimental groups included Control, Isoflurane, Isoflurane + TNF-α/IFN-γ, Isoflurane + Ferrostatin-1, Isoflurane + zVAD-fmk, Isoflurane + Ferrostatin-1 + zVAD-fmk, and Isoflurane + TNF-α/IFN-γ + inflammatory neutralization treatment. Exact n for each assay was as follows: for panels B–F, n = 4 independent cultures/group. Unless otherwise specified, data are shown as mean ± SD. Statistical analyses were performed as described in Section 2.9.

As shown in Figure 8, an expanded *in vitro* functional module was performed to test whether immune-associated signaling contributes to suppression of the astrocytic SLC7A11/GPX4 defense axis after isoflurane exposure and to compare ferroptosis-associated and apoptosis-associated injury components. Relative to the isoflurane group, combined TNF-α/IFN-γ challenge further reduced SLC7A11 and GPX4 expression and increased ACSL4 and PTGS2 levels (Figure 8B), indicating that inflammatory stimulation aggravated disruption of the astrocytic antioxidant defense program.

This inflammatory aggravation was accompanied by greater lipid ROS accumulation, higher 4-HNE burden, lower glutathione levels, and increased Fe^2+^ accumulation (Figures 8C,D), supporting the interpretation that immune-associated cues intensify ferroptosis-related redox failure in exposed astrocytes. In parallel, inflammatory challenge also increased cleaved caspase-3 and cleaved PARP1 levels and further reduced cell viability (Figures 8E,F), indicating that apoptosis-associated injury signaling was also enhanced under these conditions.

To clarify pathway contribution, Ferrostatin-1 and zVAD-fmk were compared directly. Ferrostatin-1 more effectively improved lipid peroxidation-associated and ferroptosis-associated readouts, including lipid ROS, 4-HNE, GSH depletion, Fe^2+^ accumulation, ACSL4, and PTGS2, whereas zVAD-fmk more strongly reduced cleaved caspase-3 and cleaved PARP1. The combined Ferrostatin-1 + zVAD-fmk condition produced the greatest overall rescue across molecular injury markers and cell viability, indicating that ferroptotic and apoptotic components coexist and can be partially suppressed in parallel.The quantitative endpoint-level values for inflammatory aggravation, inflammatory neutralization, and pathway-comparison rescue are summarized in Table 7.

**TABLE 7:** Quantitative summary of inflammatory challenge, inflammatory neutralization, and pathway-comparison rescue in primary astrocytes.

Endpoint	Control	Isoflurane	Isoflurane + TNF-α/IFN-γ	Isoflurane + Fer-1	Isoflurane + zVAD-fmk	Isoflurane + Fer-1 + zVAD-fmk	Isoflurane + TNF-α/IFN-γ + neutralization
SLC7A11 protein (rel. to β-actin)	1.00 ± 0.10	0.61 ± 0.08	0.42 ± 0.07	0.81 ± 0.09	0.66 ± 0.08	0.86 ± 0.08	0.74 ± 0.09
GPX4 protein (rel. to β-actin)	1.00 ± 0.09	0.68 ± 0.07	0.49 ± 0.06	0.84 ± 0.08	0.71 ± 0.07	0.88 ± 0.07	0.79 ± 0.08
ACSL4 protein (rel. to β-actin)	1.00 ± 0.11	1.39 ± 0.14	1.71 ± 0.18	1.12 ± 0.13	1.31 ± 0.15	1.05 ± 0.12	1.21 ± 0.13
PTGS2 mRNA (fold)	1.00 ± 0.16	1.84 ± 0.24	2.31 ± 0.28	1.28 ± 0.20	1.72 ± 0.22	1.19 ± 0.18	1.44 ± 0.21
Lipid ROS (C11-BODIPY ox/reduced ratio)	1.00 ± 0.12	1.82 ± 0.19	2.27 ± 0.23	1.24 ± 0.15	1.67 ± 0.18	1.13 ± 0.14	1.41 ± 0.17
4-HNE IF intensity (AU)	1.00 ± 0.11	1.63 ± 0.18	2.01 ± 0.21	1.19 ± 0.14	1.51 ± 0.17	1.11 ± 0.13	1.33 ± 0.15
GSH (μmol/g protein eq.)	5.81 ± 0.52	4.19 ± 0.41	3.41 ± 0.39	5.17 ± 0.46	4.42 ± 0.43	5.34 ± 0.44	4.88 ± 0.45
Fe2+ (μg/g protein eq.)	17.9 ± 2.4	23.8 ± 2.8	27.2 ± 3.1	19.8 ± 2.5	22.7 ± 2.7	19.1 ± 2.4	21.1 ± 2.6
Cleaved caspase-3 (rel. to β-actin)	1.00 ± 0.12	1.71 ± 0.18	2.02 ± 0.20	1.33 ± 0.15	1.08 ± 0.13	0.96 ± 0.11	1.39 ± 0.16
Cleaved PARP1 (rel. to β-actin)	1.00 ± 0.10	1.62 ± 0.17	1.91 ± 0.19	1.29 ± 0.14	1.04 ± 0.11	0.93 ± 0.10	1.31 ± 0.14
Cell viability (% of control)	100.0 ± 5.2	69.4 ± 6.8	54.8 ± 6.2	84.1 ± 7.1	78.3 ± 6.7	90.6 ± 6.3	80.2 ± 6.9

Values are reported as mean ± SD from independent primary astrocyte cultures. Data summarize endpoint classes used to compare inflammatory aggravation, inflammatory neutralization, and differential rescue by Ferrostatin-1 and zVAD-fmk. Lipid ROS, 4-HNE, glutathione-related readouts, Fe^2+^, ACSL4, and PTGS2 were interpreted as ferroptosis-associated or lipid peroxidation-associated indicators, whereas cleaved caspase-3 and cleaved PARP1 were interpreted as apoptosis-associated indicators. These results provide functional support for inflammatory contribution to astrocytic SLC7A11/GPX4 suppression but do not replace direct genetic perturbation experiments.

Importantly, inflammatory neutralization partially restored SLC7A11 and GPX4 expression and attenuated downstream redox and lipid peroxidation abnormalities in cytokine-challenged astrocytes (Figures 8B–D). Although this rescue was incomplete, it provides functional support for a model in which immune-associated signaling contributes to suppression of the astrocytic SLC7A11/GPX4 axis rather than merely co-occurring with it.

Together, these data refine the mechanistic interpretation of the astrocyte injury state after isoflurane exposure. Specifically, they support a mixed injury-state framework in which inflammatory signaling functionally contributes to astrocytic SLC7A11/GPX4 suppression, ferroptosis represents a prominent but non-exclusive component of the injury phenotype, and apoptosis-associated signaling coexists in parallel rather than serving as a mutually exclusive alternative pathway.

## 4 Discussion

This study examined neonatal isoflurane-associated neurotoxicity using an integrated framework combining single-cell and bulk transcriptomics with *in vivo* and *in vitro* validation, and the findings support a glia-centered neuroimmune ferroptosis-associated vulnerability framework (Zhao et al., 2024). Rather than representing an exclusively neuron-centered injury process, the data indicate that early anesthetic exposure is associated with remodeling of the glia-regulated neuroimmune microenvironment, accompanied by increased lipid peroxidation burden, inflammatory activation, and attenuation of antioxidant defense programs. These changes were observed in parallel with neurodevelopment-related phenotypes, including impaired hippocampal neurogenesis and altered cognitive-behavioral outcomes. Taken together, the findings provide a coherent basis for understanding why neonatal developmental windows may be especially sensitive to anesthetic-associated injury, particularly when early cell-state stress responses are engaged (Chen et al., 2025).

From a pathobiological perspective, ferroptosis is a biologically plausible and consistently supported component of this injury state. Ferroptosis is characterized by iron-dependent membrane lipid peroxidation and is constrained by glutathione-dependent antioxidant buffering and GPX4-mediated detoxification of lipid peroxides (Deng et al., 2025). In the present study, convergent evidence from transcriptomic, biochemical, histological, and cellular assays showed increased lipid peroxidation, impaired redox reserve, and reduced axis-associated antioxidant defense after isoflurane exposure. At the same time, the expanded pathway-comparison experiments showed that apoptosis-associated signaling was also involved. Ferrostatin-1 more effectively improved lipid peroxidation-associated and ferroptosis-associated readouts, whereas zVAD-fmk more strongly reduced cleaved caspase-3 and cleaved PARP1, and the combined intervention produced the greatest overall rescue. This pattern supports a mixed injury-state interpretation in which ferroptosis-associated vulnerability is prominent but coexists with apoptosis-associated and other stress-response signaling rather than acting as an isolated mechanism.

The molecular focus of this study was the SLC7A11/GPX4 antioxidant defense axis, which links cystine uptake, glutathione synthesis, and lipid peroxide detoxification. Functional suppression of this axis would be expected to weaken cellular resistance to lipid oxidative damage, particularly under inflammatory and iron-handling stress (Sun et al., 2025). The present findings consistently identified SLC7A11/GPX4-associated changes as a prominent feature of the observed redox and lipid peroxidation phenotype across multiple data layers. Importantly, the additional inflammatory challenge and neutralization experiments extended the correlative multi-omics observations by providing functional support that immune-associated signaling contributes to suppression of the astrocytic SLC7A11/GPX4 defense axis. In cytokine-challenged astrocytes, TNF-α/IFN-γ further aggravated axis suppression, lipid ROS accumulation, glutathione depletion, iron dysregulation, and downstream injury burden, whereas inflammatory neutralization partially restored SLC7A11/GPX4-related readouts. These findings strengthen the interpretation that neuroimmune remodeling is not merely concurrent with ferroptosis-associated vulnerability but may actively lower the threshold for astrocytic redox failure after isoflurane exposure.

At the same time, the strength of this conclusion should be interpreted in light of the intervention strategy used. Ferrostatin-1 provides ferroptosis-associated pharmacological support, NAC plus selenium supports antioxidant and selenoprotein-dependent defense capacity, and zVAD-fmk suppresses caspase-dependent apoptotic signaling, but none of these approaches substitutes for direct genetic perturbation of SLC7A11/GPX4 in defined glial populations. Accordingly, the intervention results are best interpreted as evidence that strengthening ferroptosis-related antioxidant defense and reducing coexisting apoptotic stress are associated with partial reversal of injury phenotypes, rather than as definitive proof that SLC7A11/GPX4 disruption alone is sufficient to drive the entire phenotype.

At the cellular level, single-cell analyses identified astrocytes and microglia as the most prominent glial populations associated with the neuroimmune ferroptosis-associated vulnerability pattern (Li Y. et al., 2025). Astrocytes showed reduced expression of system Xc^−^/glutathione/GPX4-associated defense genes together with increased stress-associated features, whereas microglia exhibited inflammatory activation and coordinated changes in iron-handling and lipid-stress pathways. These parallel glial programs provide a plausible basis for local amplification of oxidative and inflammatory injury signals. The additional astrocyte-focused functional experiments are consistent with this interpretation by showing that inflammatory stimulation can further aggravate astrocytic axis suppression and redox failure under isoflurane-associated conditions. In addition, macrophage/monocyte-like cells increased after exposure, which may reflect broader neuroimmune remodeling; however, this population remained relatively small and should be interpreted cautiously until validated using more specific lineage-resolving approaches. Because the scRNA-seq libraries were generated from pooled hippocampal and cortical tissue, these cell-state findings should also be interpreted as cross-region neonatal brain trends rather than region-specific conclusions.

Several limitations should be noted. First, the pooled cortex-plus-hippocampus single-cell design improved cell yield and broad atlas coverage but limited regional resolution of transcriptomic responses. Region-specific validation, such as separate hippocampal and cortical profiling or spatially resolved assays, will be important in future work. Second, although the pharmacological rescue results, inflammatory challenge/neutralization experiments, and mediation-based indirect-effect models support pathway-level consistency and provide functional reinforcement, they do not replace direct genetic gain- and loss-of-function experiments targeting SLC7A11/GPX4 in defined glial populations. Third, the rescue effects were partial, which is consistent with a multi-pathway injury process and further supports the interpretation that ferroptosis-associated vulnerability is prominent but not exclusive. Finally, longer follow-up and dose-duration stratification studies are needed to determine the durability, safety window, and translational feasibility of peri-anesthetic neuroprotective strategies (Wang et al., 2024; Mohan et al., 2024; Wang et al., 2023).

Despite these limitations, the present study provides convergent evidence across molecular, cellular, tissue, and behavioral levels supporting a glia-centered neuroimmune ferroptosis-associated vulnerability framework after neonatal isoflurane exposure. More specifically, the findings support a model in which neuroimmune activation functionally contributes to suppression of astrocytic SLC7A11/GPX4-associated antioxidant defense, thereby promoting a redox-vulnerable state in which ferroptosis-related and apoptosis-related injury programs can coexist. This framework offers a practical basis for future mechanistic refinement and for developing peri-anesthetic neuroprotection strategies that target redox defense and lipid peroxidation without altering the anesthetic procedure itself.

## 5 Conclusion

Neonatal isoflurane exposure is associated with a neurotoxicity phenotype that extends beyond neuron-centered injury and involves broader remodeling of the glia-regulated neuroimmune microenvironment (Zhang et al., 2023; Liu et al., 2021). Across integrated transcriptomic, *in vivo*, and *in vitro* analyses, the findings consistently support a ferroptosis-associated vulnerability state characterized by increased lipid peroxidation, inflammatory activation, and reduced SLC7A11/GPX4-associated antioxidant defense (Li R. et al., 2025).

Additional functional experiments further indicate that immune-associated signaling contributes to suppression of the astrocytic SLC7A11/GPX4 defense axis and aggravates downstream redox and lipid peroxidation injury. Pharmacological rescue experiments further indicate that this injury-associated vulnerability state is at least partially reversible: intervention was associated with restoration of axis-related markers, reduced lipid peroxidation burden, and improvement in selected cellular, tissue, and behavioral readouts. At the same time, differential rescue patterns across Ferrostatin-1 and zVAD-fmk conditions indicate that the injury program is mixed rather than exclusively ferroptotic, with ferroptosis representing a prominent but non-exclusive component.

Overall, this study supports a glia-centered neuroimmune ferroptosis-associated vulnerability framework for neonatal isoflurane-associated brain injury and identifies the SLC7A11/GPX4 axis as a candidate intervention-relevant node for peri-anesthetic neuroprotection during critical stages of brain development (Mashayekhi et al., 2025). Further genetic and region-specific validation will be needed to define the precise causal hierarchy of this pathway in the developing brain.

## Data Availability

The data presented in this study are deposited in the NCBI Gene Expression Omnibus (GEO) repository, accession numbers GSE275841 and GSE275842. The single-cell RNA-seq dataset is available under accession number GSE275841, and the bulk RNA-seq dataset is available under accession number GSE275842. Processed data underlying the main figures and tables are included in the article and Supplementary Material. Additional processed data, analysis metadata, and code or parameter information supporting the findings of this study are available from the corresponding author upon reasonable request.
